# Cabozantinib Response in a Patient With NSCLC Harboring Both MET Exon 14 Skipping Mutation and Secondary RET Fusion: A Case Report

**DOI:** 10.1016/j.jtocrr.2024.100647

**Published:** 2024-02-07

**Authors:** Carlos Torrado, Jamie Feng, Elizabeth Faour, Natasha B. Leighl

**Affiliations:** a Medical Oncology Department, Hospital Universitario Virgen del Rocío, Seville, Spain; b Princess Margaret Cancer Centre, University Health Network, Toronto, Ontario, Canada

**Keywords:** MET exon 14, RET fusion, TKI, Non–small cell lung cancer, Case report

## Abstract

MET exon 14 skipping mutation has emerged as a new oncogenic driver in NSCLC with available targeted therapies, including Food and Drug Administration–approved inhibitors capmatinib and tepotinib. Potential resistance mechanisms are beginning to be described and include several on-target and off-target mutations. Here, we report an emergent secondary RET fusion in a patient with a primary MET exon 14 skipping mutation that progressed on capmatinib after the initial response. Subsequently, this patient received both a RET inhibitor (selpercatinib) followed by another MET-targeted treatment (tepotinib) without clinical benefit. Thereafter, cabozantinib, a multikinase inhibitor with activity against RET and MET was started with a rapid clinical and radiologic benefit.

## Introduction

Co-occurring driver mutations are found in approximately 1.5% of patients with NSCLC.^1^ However, the emergence of off-target secondary mutations after treatment with tyrosine-kinase inhibitors (TKI) in patients with primary mutant NSCLC is a common resistance mechanism, resulting in co-occurring driver mutations.

MET resistance mechanisms after TKIs include on-target (35%) and off-target (45%) mutations and unknown mechanisms in 25% of cases. The most common off-target secondary resistance mechanisms are acquired KRAS and EGFR mutations.^2^ The emergence of a RET fusion mutation after TKI treatment has not been reported in primary mutant MET exon 14 skipping mutation NSCLC, although it has been described in other driver mutation–positive lung cancers.^3^

Capmatinib and tepotinib are targeted therapies that have been recently approved for MET exon 14 skipping mutation NSCLC in the second or further lines. Similarly, selpercatinib and pralsetinib have been approved for RET fusion NSCLC. However, the optimal treatment of patients with concurrent MET and RET fusion mutations is yet to be defined in this rare subgroup.

## Materials and Methods

The patient involved in this case report gave their informed consent authorizing the use and disclosure of their health information. The chart was reviewed for patient details, pathological findings, and treatment details. Response to treatment was assessed using Response Evaluation Criteria in Solid Tumors version 1.1.

## Case Presentation

**Figure 1:**
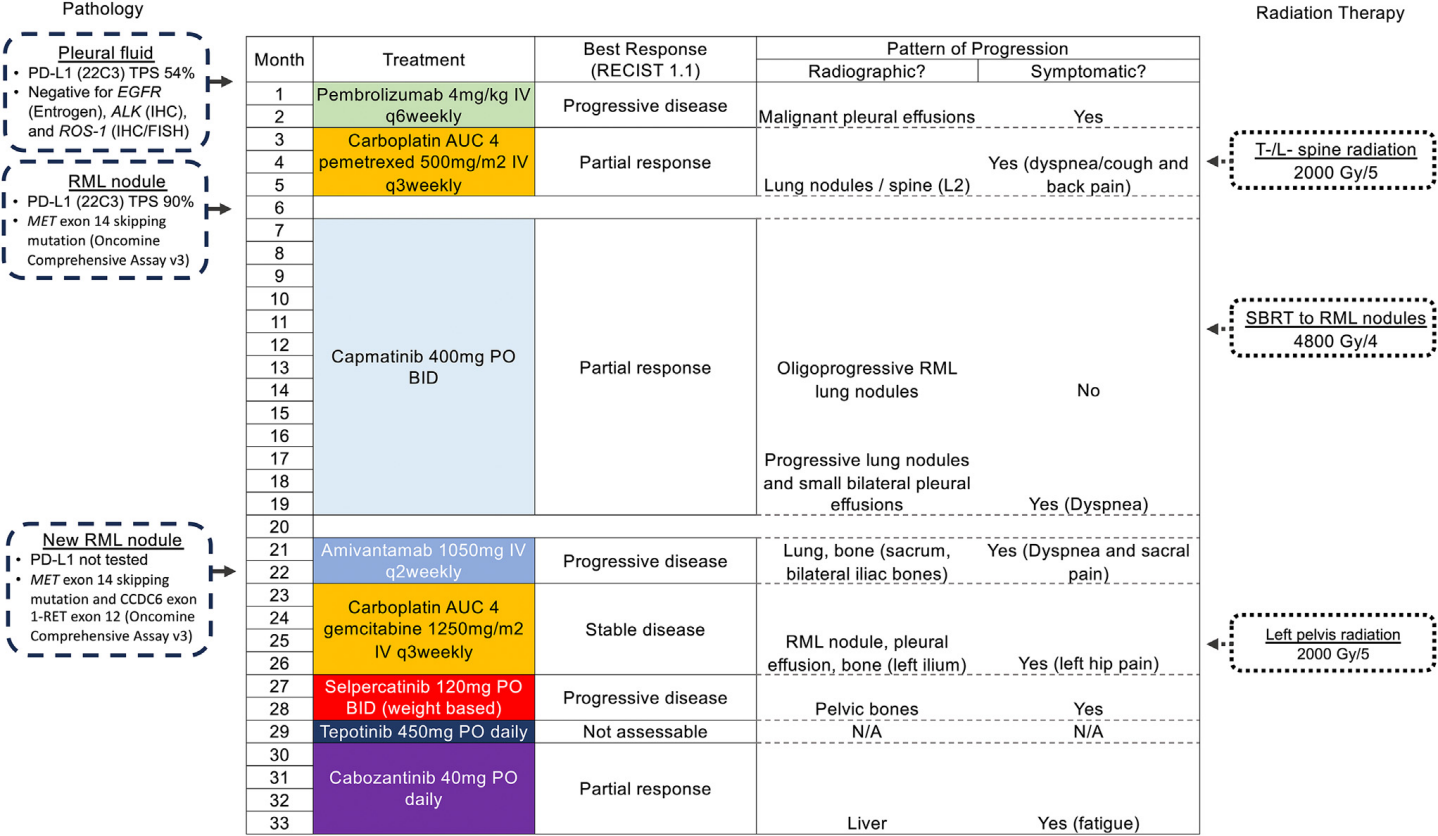
Visual summary of treatment timeline. AUC, area under the curve; BID, twice daily; FISH, fluorescence in situ hybridization; IHC, immunohistochemistry; IV, intravenous; N/A, not applicable; PD-L1, programmed death-ligand 1; PO, orally; q3 weekly, every 3 weeks; q6 weekly, every 6 weeks; RECIST, Response Evaluation Criteria in Solid Tumors; RML, right middle lobe; SBRT, stereotactic body radiotherapy; T/L, thoracolumbar; TPS, tumor proportion score; v, version.

We describe a case of a lifetime nonsmoker Asian 70-year-old woman with no baseline comorbidities or past medical history, diagnosed with metastatic lung adenocarcinoma with pleural and bone metastases. The Eastern Cooperative Oncology Group Performance Status was 1 at the time of diagnosis. The patient’s treatment timeline is visually summarized in Figure 1. Molecular biomarkers on the initial pleural fluid were negative for EGFR (Entrogen, Los Angeles, CA), ALK (immunohistochemistry), and ROS1 (immunohistochemistry and fluorescence in situ hybridization). The programmed death-ligand 1 tumor proportion score on this sample was 54%. The patient received pembrolizumab as a first-line treatment; however, she progressed after two cycles with worsening dyspnea because of progressive malignant effusions. Carboplatin-pemetrexed as a second-line treatment achieved an initial partial response lasting only 4 months.

**Figure 2:**
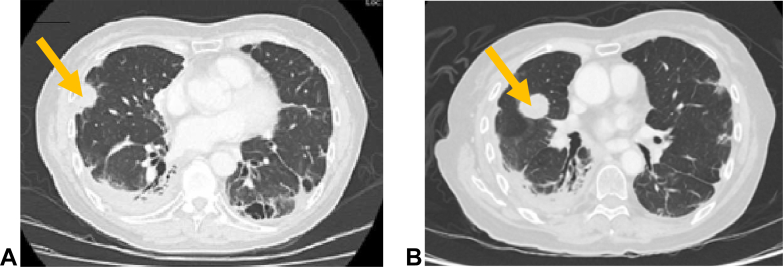
(*A*) CT scan (lung window) July 2021 illustrating a new 25-mm RML lateral nodule on chemotherapy that was biopsied revealing MET exon 14 skipping mutation. (*B*) CT scan (lung window) May 2022 illustrating a new 23 by 31 mm RML lesion on amivantamab that was subsequently biopsied to reveal the concurrent RET fusion. CT, computed tomography; RML, right middle lobe.

Biopsy of a right middle lobe lesion (Fig. 2*A*) revealed a poorly differentiated NSCLC favoring adenocarcinoma. Molecular testing with Oncomine Comprehensive Assay version 3 next-generation sequencing (able to detect single nucleotide variants, small insertions and deletions, copy number variants, and gene fusions) revealed a MET exon 14 skipping mutation. Capmatinib was started at 400 mg orally twice daily and a partial response was documented radiographically at 2 months. Two oligoprogressive lung lesions were treated with stereotactic body radiotherapy at 7 months after initiation of capmatinib; however, ongoing multifocal progression led to the discontinuation of capmatinib after a total of 13 months of therapy. Fourth-line amivantamab did not confer clinical benefit and the patient had progressive disease. Biopsy of a new right middle lobe lesion (Fig. 2*B*) was taken, and, using the same Oncomine next-generation sequencing panel (ThermoFisher Scientific, Waltham, MA) as the previous biopsy, persistence of the MET exon 14 skipping mutation was exhibited; but this time, a treatment-emergent CCDC6 exon 1-RET exon 12 fusion was also identified. Both were detected with a read count of greater than 2000 as was the minimum for detection with the assay. The patient was started on gemcitabine and carboplatin combination chemotherapy, on which she progressed after 5 cycles both clinically (increasing bone pain) and radiographically (increase in the size of a right lower lobe nodule and pleural effusion; however, these did not meet the criteria for measurable disease progression). Because of patient preference and the combination of clinical and radiographic findings concerning progression, she was started on sixth-line treatment with selpercatinib. On selpercatinib, she had ongoing bone pain requiring close follow-up by the palliative care team and palliative radiation. She developed dyspnea and cough accompanied by radiographic evidence of progression in the lungs. Tepotinib was chosen as seventh-line therapy but discontinued after 2 weeks because of poor tolerance (grade 2 nausea, grade 3 asthenia) and minimal symptomatic benefit with ongoing dyspnea, but improved left hip pain. This improvement may have been from tepotinib, but is confounded by palliative radiation to her pelvis. Formal radiographic restaging was not performed after tepotinib as she had only been on treatment for 1 month.

**Figure 3:**
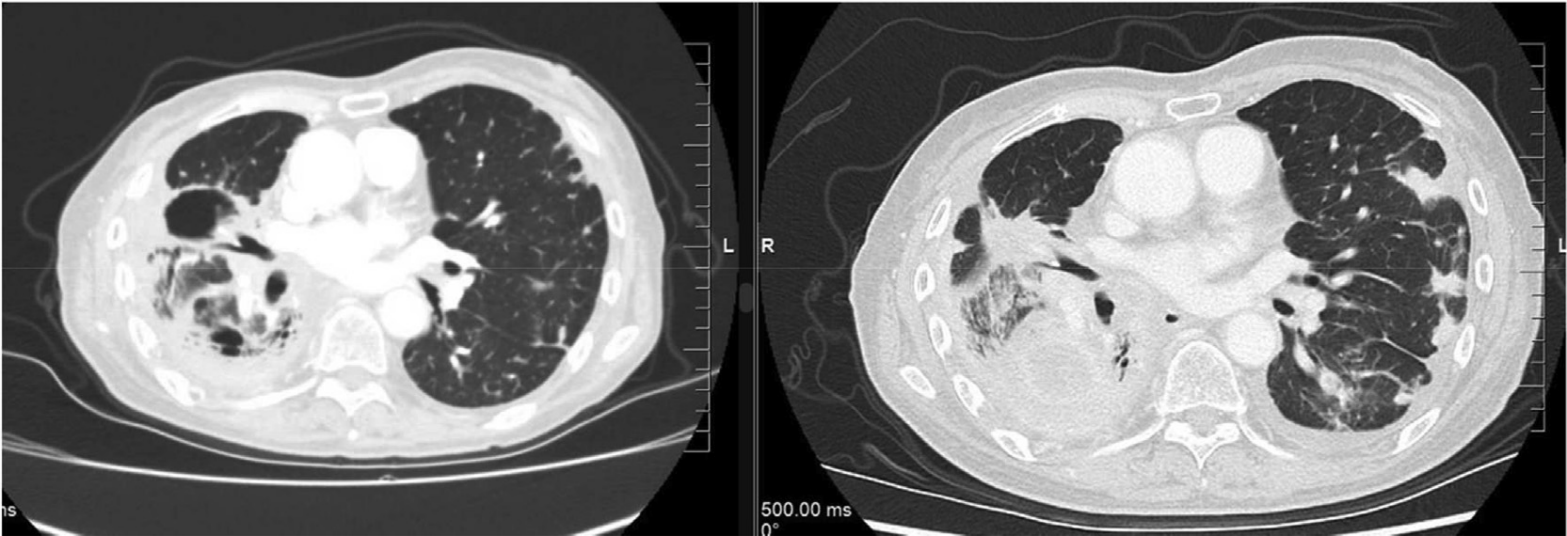
CT slice illustrating improvement in the right midlung lesions and left lung peripheral lesions after 6 weeks of cabozantinib (left) compared with a similar CT slice from 2 weeks before starting (right). CT, computed tomography.

Cabozantinib 40 mg oral daily was started as eighth-line therapy 30 months after the initial cancer diagnosis. The extent of the disease at that time continued to be limited to the lungs, pleura, and bones. After 2 weeks of cabozantinib, the patient experienced improved hip pain, resolution of dyspnea, and no adverse effects. The symptomatic improvement was so profound that the patient decided to abandon a medically assisted death request she had made 2 weeks before. A computed tomography scan after 6 weeks revealed a partial response initially (Fig. 3) but she progressed systemically with new liver metastases and died 4.5 months after starting cabozantinib.

## Discussion

To our knowledge, this is the first report of a RET fusion as a potential secondary resistance mechanism after capmatinib in a patient with MET exon 14 skipping mutant lung cancer. Conversely, MET dysregulation has been described as a resistance mechanism in 15% of patients with RET fusion–positive NSCLC treated with selective RET TKIs.^4^ Tumor heterogeneity leading to a subclonal event and emergent RET fusion could have led to progressive disease on MET-targeted treatment.

After the detection of the RET fusion, the patient did not respond to targeted therapies for either MET or RET alone. Interestingly, cabozantinib achieved a rapid clinical and radiologic response in this co-mutated patient, albeit not durable. Cabozantinib is known to have activity against MET, RET, vascular epithelial growth factor, KIT, AXL, and FLT3 receptors, and has an overall response rate of 28% in RET fusion NSCLC.^5^ It may be that inhibition of both oncogenic pathways was needed to achieve a response.

In the reverse molecular scenario, dual blockade also achieved a response. In a primary RET fusion NSCLC with an acquired MET amplification on selpercatinib, the addition of the multikinase inhibitor crizotinib overcame TKI resistance in four patients.^4^ These data suggest that patients with concurrent MET alterations and RET fusions may require inhibition of both pathways.

A limitation of this study is the short exposure to tepotinib resulting in a lack of definite evidence that the patient’s disease would not have been controlled on this drug. In addition, whereas dual blockade of MET and RET pathways is one plausible explanation for the patient’s dramatic response, there are alternative hypotheses. Cabozantinib has antiangiogenic properties, which may have contributed to the dramatic response.

## Conclusion

We describe a case of lung adenocarcinoma with MET exon 14 skipping mutation and an emergent secondary RET fusion after treatment and initial response to the MET inhibitor capmatinib. After detection of the RET fusion, treatment with selpercatinib and tepotinib did not lead to a response. Interestingly, the multikinase inhibitor cabozantinib provoked a rapid clinical and radiologic response. This case suggests that less selective TKIs may provide increased benefit in patients with more than one targetable mutation in NSCLC.

## CRediT Authorship Contribution Statement

**Carlos Torrado:** Conceptualization; Investigation; Visualization; Roles/Writing - original draft; Writing - review & editing.

**Jamie Feng:** Conceptualization; Data curation; Investigation; Formal analysis; Visualization; Roles/Writing - original draft; Writing - review & editing.

**Elizabeth Faour:** Conceptualization; Data curation; Investigation; Formal analysis; Visualization; Roles/Writing - original draft; Writing - review & editing.

**Natasha Leighl:** Conceptualization; Supervision; Writing - review & editing.

## Disclosure

Dr. Feng reports receiving honoraria as a speaker for AstraZeneca Canada. Dr. Leighl reports receiving research funding through institution from 10.13039/100002429Amgen, 10.13039/100008207AstraZeneca Canada, 10.13039/100004326Bayer, 10.13039/100004755EMD Serono, Guardant Health, 10.13039/100019518Eli Lilly, Merck Sharp & Dohme Oncology, 10.13039/100021981Roche Canada, and 10.13039/100016040Takeda; and received funding for travel, accommodations, and other expenses from Merck Sharp & Dohme. The remaining authors declare no conflict of interest.

## References

[1] Zhao Y., Wang S., Yang Z. (2021). Co-occurring potentially actionable oncogenic drivers in non-small cell lung cancer. Front Oncol.

[2] Recondo G., Bahcall M., Spurr L.F. (2020). Molecular mechanisms of acquired resistance to MET tyrosine kinase inhibitors in patients with MET Exon 14–mutant NSCLC. Clin Cancer Res.

[3] Wang C., Zhang Z., Sun Y. (2022). RET fusions as primary oncogenic drivers and secondary acquired resistance to EGFR tyrosine kinase inhibitors in patients with non-small-cell lung cancer. J Transl Med.

[4] Rosen E.Y., Johnson M.L., Clifford S.E. (2021). Overcoming MET-dependent resistance to selective RET inhibition in patients with RET fusion-positive lung cancer by combining selpercatinib with crizotinib. Clin Cancer Res.

[5] Drilon A., Rekhtman N., Arcila M. (2016). Cabozantinib in patients with advanced RET-rearranged non-small-cell lung cancer: an open-label, single-centre, phase 2, single-arm trial. Lancet Oncol.

